# Precocity in a Boy

**Published:** 1841-01

**Authors:** 


*Precocity in a Boy.*—The case of precocious puberty in a female child mentioned in your Journal of Dec. 2, reminded me of quite a curious instance in a boy now living with his parents in this city.

William S. W*** was five years old on the 9th of last August. At his birth he weighed eight pounds. During the first twelve months he did not grow with very remarkable rapidity; at eighteen months he had become uncommonly large, and has ever since continued to grow rapidly. He began to walk when one year old, and to talk distinctly about a year later. Until within a few months he has been sprightly and energetic in all his movements. During the summer past he has lost several pounds of flesh, and has seemed feeble and reluctant to engage in his ordinary amusements. He has never been very fat; on the contrary, rather lean. His appetite, until he was two and a half years old, was delicate, and milk was his principal nourishment; after that period he began to relish other kinds of food and to eat largely, as much so at times as an ordinary laboring man. The parents suspect, that not having been with them during the past summer, the boy has suffered for want of a proper allowance of nutritious food, and to the latter circumstance, chiefly, they attribute his loss of flesh and activity. At the age of four years the child weighed sixty pounds avoirdupois; and when he was five years old his weight was seventy pounds, although, as his father stated, "he was quite thin at the time." His height is four feet six inches. His body is rather long in proportion to his limbs; when his health is good, his muscular strengh is proportionate to his size.

The organs of generation began to exhibit an unusual development as early as the eighteenth month and are now nearly of the adult size. There is a trifling growth of hair on the pubes. There is no evidence of excessive sexual desire, although on this point the parents are unable to speak decidedly. His voice for more than a year has been on the bass key.

He has habits of quick and accurate observation; his memory is retentive, and his mind of an inquisitive cast. He reads fluently in easy lessons, but no efforts of any description have been made to draw out his mental powers. His head is large: and, regarded phrenologically or otherwise, is well shaped. He is readily moved to tears—and injury produces grief rather than ager. His resentments, when they do arise, are rather sudden than lasting. Occasionally he indulges in bursts of mirth, but commonly his deportment is grave, with a prevailing air of melancholy.

In all the peculiarities of the boy there is a close resemblance to the early history of the father, who is now about thirty-five years of age, and who acquired his present stature at the age of *nine years*. The family from which the father's mother sprung has presented several instances of similar precocity, all of which occurred in the *male* descendants. In these instances there was uniformily the same peculiar conformation and want of symmetrical figure, produced by a long trunk surmounted upon short limbs.

There is one other circumstance connected with the ancestry of this lad, which although not absolutely pertinent and necessary to be related here, is exceedingly interesting to me, and I dare say will be so to a majority of your readers. I am induced to mention it, because it may serve as a key to modulate our expectations in regard to the future destiny of the child. The father is one of the ripest and most accomplished scholars in New England, and has for several years been distinguished and publicly praised for his poetical talents; and before he reached his twenty-third year he wrote a learned essay, which, although opposed by several rivals, won a premium offered for the best production on the given subject. The father's mother was likewise remarkable for intellectual endowments, and one rare attribute of her mind was that whatever she read was retained in her memory like a nail driven in a sure place.—*Boston Med. and Sur. Jour. for Dec.* 1840.

